# New Potassium Sodium Niobate Single Crystal with Thickness-independent High-performance for Photoacoustic Angiography of Atherosclerotic Lesion

**DOI:** 10.1038/srep39679

**Published:** 2016-12-21

**Authors:** Benpeng Zhu, Yuhang Zhu, Jie Yang, Jun Ou-Yang, Xiaofei Yang, Yongxiang Li, Wei Wei

**Affiliations:** 1 School of Optical and Electronic Information, Huazhong University of Science and Technology, Wuhan 430074, China; 2 Key Laboratory of Inorganic Functional Materials and Devices, Chinese Academy of Sciences, Shanghai 200050, China; 3 Hubei Cancer Hospital, Wuhan 430079, China

## Abstract

The synthesis of (K_0.45_Na_0.55_)_0.96_Li_0.04_NbO_3_ (KNLN) single crystals with a <100>-orientation, using a seed-free solid state crystal growth method, is described here. With the thickness of the crystals decreasing down to the order of tens of micrometers, this new lead-free single crystal exhibits thickness-independent electrical behavior, and maintains superior piezoelectric constant (*d*_*33*_ = 670 pC N^−1^) and electromechanical coupling factor (*k*_*t*_ = 0.55). The successful fabrication of a tiny intravascular photoacoustic probe, with a 1 mm outside diameter, is achieved using a single crystal with a thickness of around 60 μm, in combination with a 200 μm core multimode fiber. Wire phantom photoacoustic images show that the axial resolution and lateral resolution of the single crystal based probe are 60 and 220 μm, respectively. In addition, intravascular photoacoustic imaging of the atherosclerotic lesion of a human artery is presented. In the time-domain and frequency-domain images, calcified regions are clearly distinguishable from surrounding tissue. These interesting results demonstrate that KNN-based lead-free piezoelectric single crystals are a promising candidate to substitute for lead-based piezoelectric materials for photoacoustic imaging in the future.

Atherosclerosis is the leading cause of cardiovascular disease, which is one of the major risk factors of human health^1^. To meet the needs of a precise diagnosis and an optimal treatment of atherosclerosis, it is important to provide an accurate evaluation of the atherosclerotic process, which is characterized by different plaques. Ordinarily, artery calcification appears at the advanced stage of the atherosclerotic process; however, the lively debate on whether calcification is a marker of stable or unstable plaque has increased interest on this topic^2^. Nevertheless, no clinically available imaging method can reliably and accurately detect atherosclerotic calcification embedded in plaque. For example, although computed tomography (CT) possesses the ability to detect calcium in plaque^3^, its detection is of limited physiological significance and vulnerable to plaque type. Similarly, although an intravascular ultrasound (IVUS), which is a catheter-based intravascular imaging technique with good penetration depth and axial resolution, has been demonstrated to provide structural information of atherosclerotic plaque^4^,^5^,^6^, the contrast between the lipid-rich region and other soft tissue is confined^7^,^8^,^9^. To address this issue, intravascular photoacoustic (IVPA) imaging seems to be an alternative method to detect lipid pool and atherosclerotic calcification. In this hybrid imaging process, a tiny single element ultrasound transducer, integrated with a laser fiber and driven by a rotational shaft, is placed at an appropriate position inside the blood vessel to detect the photoacoustic signals inside the tissue, which were generated by the absorption of a short pulsed laser^10^,^11^,^12^. Because the proposed intravascular photoacoustic imaging process combines the advantages of high ultrasonic resolution and strong optical absorption contrast, it shows great potential for the detecting of atherosclerotic plaque^13^,^14^.

Nowadays, most IVPA research has focused on working with the pixel values of reconstructed photoacoustic (PA) images, and has paid little attention to frequency domain analyses of the PA signal. Because the frequency spectrum of the PA signal is related to the size, distribution, and concentration of the PA absorber, it is possible to obtain significant information about the structure of the PA absorbers in terms of certain spectral parameters extracted from the PA frequency spectrum^15^. Most importantly, photoacoustic spectrum analyses have already been proven to be an effective way to expose significant differences between malignant and normal tissue regions^16^. Consequently, frequency domain analyses seem to be a beneficial supplement in IVPA technology, and could be potentially used for the characterization of atherosclerotic plaque.

No matter the time-domain image reconstruction or frequency spectrum analysis, a highly sensitive miniaturized ultrasound transducer is required for the detection of intravascular photoacoustic signals. As the core part of the transducer, lead-based piezoelectric materials have been most popular for IVPA applications because of their excellent piezoelectric behavior^10^,^11^,^12^,^13^,^14^,^15^. However, in view of environmental protection and human safety issues, the use of lead is a problem due to its toxicity. Therefore, it is of urgent need to develop lead-free piezoelectric materials that can be used as intravascular PA signal receivers. Since the work of Saito *et al*. was published in Nature^17^, potassium sodium niobate (KNN) based materials have attracted a great deal of attention, in comparison to other lead free materials, owing to their extraordinary piezoelectric properties and high Curie temperature. For the purpose of substituting lead-containing materials used in practical applications, it is worth exploring KNN-based single crystals more than KNN-based ceramics, because the former ones have better piezoelectric properties due to their higher degree of orientation and grain-boundary free microstructures^18^.

In this study, a large-sized KNN-based single crystal with a <100>-orientation was grown via a seed-free solid state crystal growth (SFSSCG) method, and the nominal formula of (K_0.45_Na_0.55_)_0.96_Li_0.04_NbO_3_ is reported to have been successfully obtained. We show that this single crystal, with a large d_33_ value of 690 pC/N, exhibits thickness-independent electrical properties. Furthermore, using the synthesized KNLN single crystal, a tiny 38 MHz single element transducer, integrated with optic fiber, was fabricated for the IVPA imaging of atherosclerotic lesion in a human artery. The aim of the present study is to demonstrate the feasibility of KNN-based single crystals to be implemented in IVPA applications and photoacoustic frequency spectrum analyses to improve the detection of lipid pool and calcification.

## Results and Discussion

### Electric properties with different thicknesses

**Figure 1:**
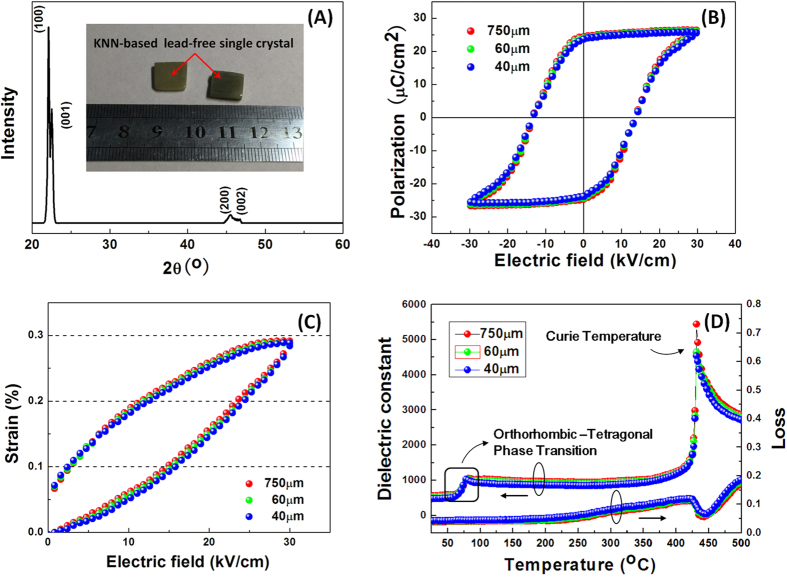
(**A**) XRD pattern of photograph of (100)-oriented KNLN lead-free single crystal; Thickness dependence of polarization hysteresis (**B**) and unipolar strain (**C**) as a function of electric field, and dielectric behavior (**D**) as temperature for this KNN-based single crystal.

Figure 1(A) presents the X-ray diffraction pattern of the crystal sample grown by the SFSSCG method. Two groups of peaks can be observed, (100) and (200), which suggest that the obtained samples, as shown in the inset, are indeed single crystals. Obviously, the (100) peak consists of two peaks, revealing that the KNN-based lead free single crystal is in the orthorhombic phase. Since an ultrasound transducer ordinarily works in the thickness mode, the device’s operational frequency is inversely proportional to the piezoelectric material’s thickness. To make sure that the KNN-based single crystal is adequate for its application as a high frequency ultrasound transducer; its thickness dependent electrical properties should be investigated. Figure 1(B) shows the P-E hysteresis loops, measured at 10 Hz and room temperature, of KNN-based single crystals with different thicknesses. As the crystal thickness decreased, the single crystal maintained its original remnant polarization (*P*_*r*_ = 24 μC/cm^2^) and coercive field (*E*_*c*_ = 13.5 kV/cm), and there was no indication of clamping/pinning of domain wall motion, even at the smallest thickness tested (40-μm). The high field piezoelectric behavior of KNN-based lead free single crystals with different thicknesses is depicted in Fig. 1(C). The effective piezoelectric coefficient (*d*_33_^*^) was deduced from the slope of the unipolar strain response (*S*_max_/*E*_max_). There was no degradation of the *d*_33_^*^ with decreasing crystal thickness, with the *d*_33_^*^ values being 970, 965, and 960 pm/V for crystal thicknesses of 750, 60 and 40 μm, respectively. These larger *d*_33_^*^ values originate from lattice distortions and switching of the non-180° domain^19^. In addition, 750 um crystal had a piezoelectric constant (*d*_33_) of 690 pC N^−1^ and 60 um crystal’s *d*_33_ slightly decreased to 674 pC N^−1^. Meanwhile, the *d*_33_ was found to remain such high level of 670 pC N^−1^, with decreasing crystal thickness down to 40 μm. This excellent piezoelectric property is superior to other KNN-based materials reported in the literatures, including non-textured ceramics, textured ceramics and single crystals^17^,^20^,^21^,^22^,^23^ and even better than PZT4 ceramics^17^. The temperature dependence of the dielectric properties of KNN-based lead free single crystals, of different thicknesses and measured at 1 kHz, are given in Fig. 1(D). In the 25–500 °C temperature range, the dielectric constant and loss of the KNN-based single crystals exhibit a nearly thickness-independent behavior. At room temperature, the values of dielectric constant/loss for crystal with thickness of 750, 60 and 40 μm, are 556/0.035, 502/0.039 and 468/0.042, respectively. Two anomalies in the dielectric constant data appeared at approximately 82 °C and 430 °C, which indicate the orthorhombic to tetragonal (O-T) and ferroelectric tetragonal to paraelectric cubic (T-C) phase-transitions, respectively. It should be noted that the high *d*_33_ of this obtained single crystal is mainly attributed to the low O-T phase transition temperature (*T*_O-T_), as has been observed for Li doped KNN systems, where low *T*_O-T_ translate into high *d*_33_ values, due to the easy polarization rotation with phase coexistence near the O-T phase boundary^24^,^25^.

**Figure 2:**
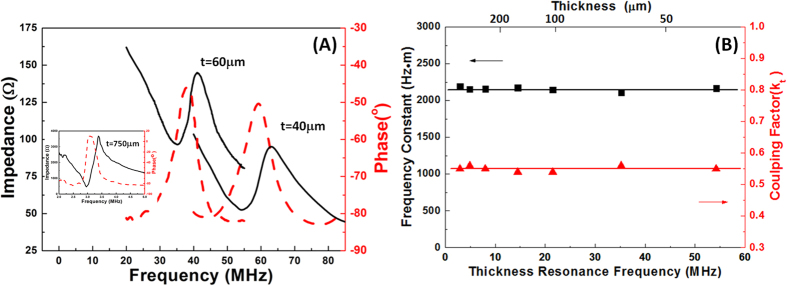
(**A**) Measured electrical impedance (solid line) and phase (dashed line); (**B**) frequency constant (N_t_) and thickness mode electromechanical coupling factor (k_t_) of KNN-based single crystal with different thickness.

As it is know, a high piezoelectric layer electromechanical coupling factor (*k*_t_) is of vital importance for ultrasound transducers because it allows for an effective energy conversion of the transmitting and receiving energy, and it improves their sensitivity of the pulse echo response. The *k*_t_ value of the KNN-based lead free piezoelectric single crystal was evaluated using the impedance method. The measured electrical impedance and phase angle of samples with different thicknesses are shown in Fig. 2(A). Even in a frequency range greater than 50 MHz, strong and clear resonance and anti-resonance peaks can be observed. The *k*_t_ of each sample as a function of frequency/thickness is presented in Fig. 2(B), the value of which is calculated according to Equation 1^26^,


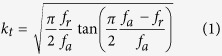


where *f*_r_ and *f*_a_ are the thickness resonance and anti-resonance frequencies, respectively. It is easily observed that the KNN-based single crystal exhibits a stable *k*_t_ value, as high as 0.55, in the frequency/thickness range studied, comparable to the *k*_t_ values of PMN-PT and PIN-PMN-PT single crystals^27^. Figure 2(B) shows the relationship between the frequency constant and sample thickness (*t*) and suggests that the frequency constant is also a stable parameter.

### IVPA Probe and Imaging

**Figure 3:**
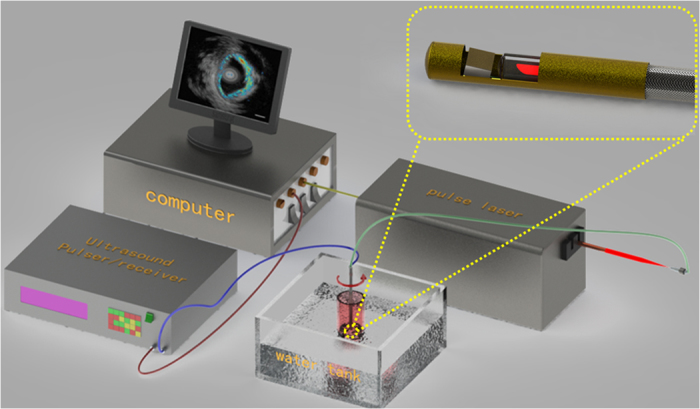
The design of intravascular photoacoustic probe and imaging system.

Figure 3 illustrates the design of the IVPA probe and imaging system. In this probe, a tiny KNLN single crystal side viewing ultrasound transducer was integrated with 200 μm core multimode fiber (MMF), to deliver a pulsed laser beam, and assembled in stainless steel tubing of a 1 mm outside diameter. The tip of the MMF was 38-deg polished, and covered with a glued on quartz capillary (ID-0.4 mm, OD-0.55 mm) to preserve an air-glass interface, and deflect the laser beam by total internal reflection. For the acoustic part, a 60 μm KNLN piezoelectric single crystal with an area of 0.4 × 0.4 mm was selected as the active element, where an E-solder 3022 was employed as the backing layer, while two matching layers were made up of a silver powder loaded epoxy and parylene, respectively. The ultrasound transducer, with a 38 MHz central-frequency and a fractional bandwidth of 56% at −6 dB, which was determined with a pulse echo test, was slightly tilted toward the fiber to achieve a maximized overlap of the optical/acoustic beam.

A tunable laser provided the probe’s excitation light (5 ns pulse width, 10 Hz repetition rate, pulse energy 1.2 mJ, OPOTEK Vibrant B/355-II). Due to the high lipid absorption at 1210 nm, the IVPA image of atherosclerotic human artery is acquired at this wavelength^13^. The free space laser output was coupled by a 4× objective lens into an optical fiber, which was then delivered to the catheter. The rotation of the distal end of the catheter was generated by a rotational motor^28^. A 5900 pulser/receiver (Olympus NDT, Inc., Kennewick, WA) was used to produce ultrasound pulses, and to receive ultrasound and photoacoustic waves. The received signals were digitized and processed in a computer. The scanning procedure was controlled by a custom-built LabVIEW program (National Instruments, Austin, TX) and synchronized by laser trigger signals.

**Figure 4:**
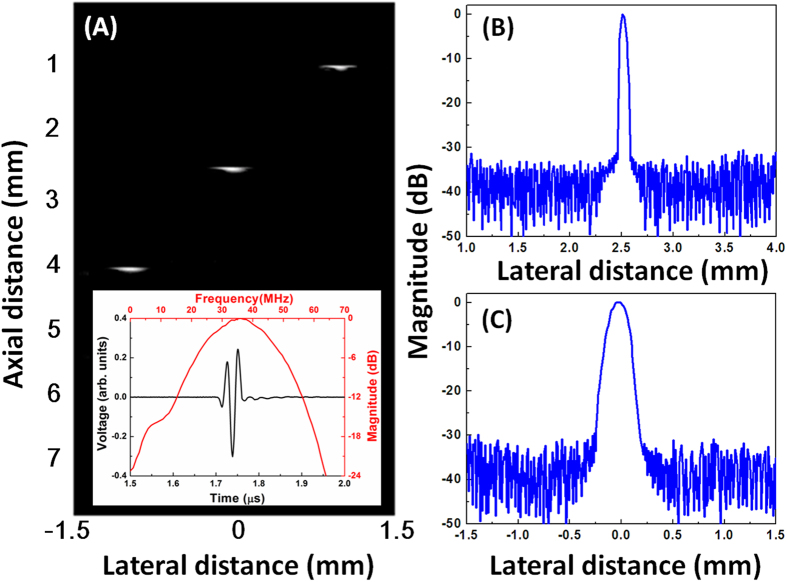
(**A**) Photoacoustic image of 25 μm tungsten wires; axial (**B**) and lateral (**C**) envelopes of the photoacoustic signal from the wire located 2.5 mm away from the transducer surface.

Firstly, to check the probe’s ability to detect PA signals, a wire phantom experiment was carried out. The photoacoustic images of the 25 μm wire targets are displayed in Fig. 4(A). The inset image shows the PA signal from a wire located at 2.5 mm away from the probe surface. Its center frequency and fractional bandwidth, at −6 dB, are 35.5 MHz and 82%, respectively. In comparison to the pulse echo test results of the KNN-based single crystal transducer, the PA signal had a much broader bandwidth, which is attributed to its broadband nature. The downshift of the central frequency is probably caused by the relatively stronger photoacoustic signals at lower frequencies. A similar phenomenon has been found when the PA signal was detected by a lead-based transducer^14^,^29^. Figure 4(B,C) present the envelopes of the photoacoustic signals from a wire located at 2.5 mm away from the probe surface. The axial resolution was 60 μm and the lateral resolution was 220 μm, which were deduced from the −6 dB envelope width.

**Figure 5:**
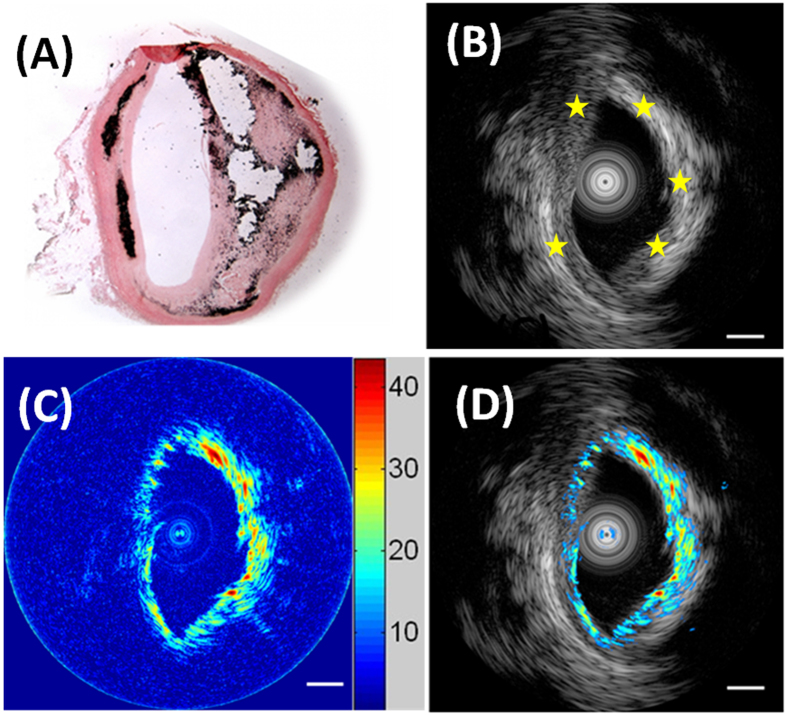
The cross-section: (**A**) Hematoxylin-Eosin (H&E) stained histology image; IVUS image (**B**) and IVPA image (**C**) of calcified atherosclerotic human artery; (**D**) Fused US and PA image of the human artery; Scale bar is 1 mm.

The histology results, shown in Fig. 5(A), indicate intimal medial thickening with an eccentric lipid-rich plaque, as well as calcified area. IVUS-imaging verifies this morphology. Typically in ultrasound images, the calcified-region embedded in a lipid pool is represented by a high ultrasound echo area among plaque, followed by a dense shadow^30^. Consequently, the calcified-regions of the human artery, marked by yellow stars in Fig. 5(B), are brighter than the surrounding lipid-rich area. As shown in Fig. 5(C), PA imaging confirms the presence of the lipid in the plaque. The PA signal of the calcification area in plaque is around 20 dB higher than the signal of the lipid, revealing that the calcification region in an IVPA image corresponds nicely to that in an IVUS image. Figure 5(D) illustrates the fused image of the structural and compositional information of the diseased human artery. These promising results indicate that the KNLN lead free piezoelectric single crystal is competent for the intravascular photoacoustic angiography of atherosclerotic lesion.

**Figure 6:**
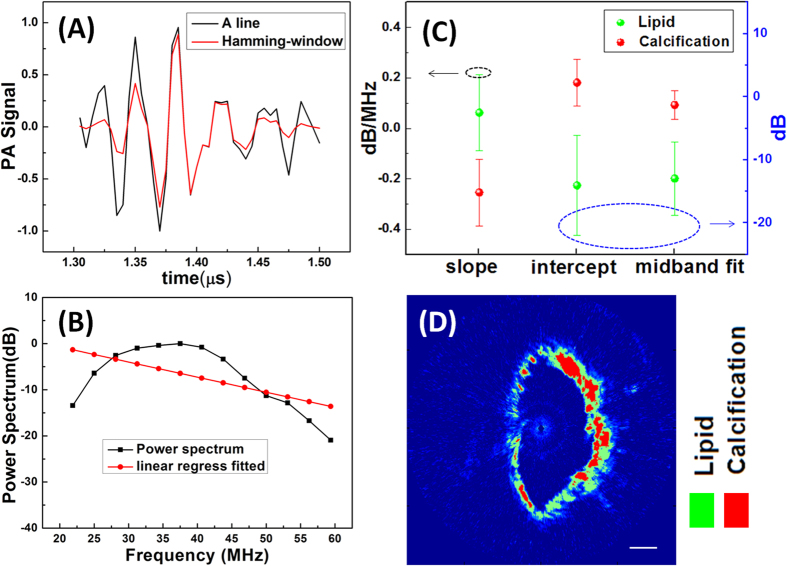
(**A**) Normalized PA A-line signal generated by the certificated region, before and after applying Hamming window; (**B**) Normalized power spectrum of windowed AP A-line fitted to linear model; (**C**) Statistical result of linear regression parameters for calcification and lipid pool; (**D**) Reconstructed image of calcified atherosclerotic human artery according to midband fit. Scale bar is 1 mm.

The beam formed digital RF data from PA imaging was imported to a MATLAB based (version. 2012a, Mathworks, Natick, MA) software for imaging data reconstruction and analysis. For comparison, the data of the calcified and lipid-rich areas were processed by the same program. The region of interest (ROI) for frequency domain analyses was chosen based on the location of the tissue in the corresponding IVPA image. Within the ROI, the RF data of each amplitude line were gated by Hamming-windows, as shown in Fig. 6(A), to minimize spectral leakage effects, and then the power spectra were calculated with a fast Fourier transform (FFT), within each gated segment, and converted to a dB scale. In Fig. 6(B), a linear regression of the power spectra was performed to generate the slope, intercept, and midband fit (the linear function evaluated at the midpoint of the usable bandwidth). Figure 6(C) shows the statistical results of PA frequency domain analysis for human atherosclerotic artery segments (n = 10). The data is plotted as mean ± standard deviation of mean value at ROIs. Both data of lipid and calcification groups meet the Shapiro–Wilk test for normality. The results demonstrate that the linear regression parameters for calcification and lipid are different, and there are significant differences in the midband fit and intercept. Afterwards, the imaging data were divided into many regions and the midband fit distributions were obtained by frequency spectrum analyses. The reconstructed image, according to the acquired midband fit, is presented in Fig. 6(D). The calcified regions and lipid pool are displayed in red and green, respectively. These results suggest that frequency domain analysis could be used to characterize atherosclerosis potentially.

## Conclusion

<100>-orientated KNN-based lead free single crystals with a nominal formula of (K_0.45_Na_0.55_)_0.96_Li_0.04_NbO_3_ were synthesized via a seed-free solid-state crystal growth method. This kind of new single crystal exhibited thickness-independent dielectric, ferroelectric, and piezoelectric behaviors. Most importantly, the obtained lead free single crystal maintained high and stable piezoelectric constants (*d*_33_ = 670 pC N^−1^), and a stable thickness mode electromechanical coupling factor (*k*_*t*_ = 0.55) at all thicknesses tested. Using a ~60-μm KNN-based single crystal with an active size of 0.4 × 0.4 mm and a 200-μm core multimode fiber, a tiny intravascular photoacoustic probe, with an outside diameter of 1 mm, was successfully fabricated. The wire phantom PA image revealed that the KNLN lead free single crystal possesses the ability to receive photoacoustic signals with high resolution. Time and frequency domain IVPA images of a human artery demonstrated that the KNLN lead free single crystal was adequate for atherosclerotic lesion angiography. These promising results suggest that the KNN-based single crystal is a good candidate to replace lead-containing materials for photoacoustic applications.

### Experimental Section

#### KNN-based single crystal preparation

To prepare (K_0.45_Na_0.55_)_0.96_Li_0.04_NbO_3_ (KNLN) single crystal using the SSCG method, a precursor powder was first prepared by the raw materials Na_2_CO_3_ (99.8%), K_2_CO_3_ (99%), Li_2_CO_3_ (98%) and Nb_2_O_5_ (99.5%). The precursor powders were uniaxially pressed into disks, following a cold isostatic press under 200 MPa. To avoid volatilization of the alkaline elements, the samples were muffled with additional pre-prepared powders in a closed alumina crucible. The details of the synthesis process and crystal growth were reported in ref. 18. KNLN single crystals with various thicknesses were prepared by lapping and polishing. Initial lapping utilized 10-μm Al_2_O_3_ powder suspended in a distilled water medium and final polishing was carried out with a 0.05-μm diamond paste to minimize surface damage which can deteriorate the material properties. Gold electrodes were deposited on sample’s both surfaces using sputtering deposition.

#### Electrical properties characterization

The dielectric constant and loss as a function of temperature and the thickness mode electromechanical coupling factors (k_t_) of the prepared samples were measured using a precision impedance analyzer (Agilent Technology, 4294 A, California, USA). For piezoelectric properties’ characterization, the crystals were poled for 30 min at room temperature under a DC electric field of 30 kV/cm. After aging for 24 h, the piezoelectric coefficient *d*_33_ was acquired using a Berlincourt-type quasi-static *d*_33_ meter (ZJ-3 A, Institute of Acoustics, Beijing, China). Polarization versus electric field (*P*–*E* loops) and unipolar strain were measured at a frequency of 10 Hz using a modified Sawyer-Tower circuit and linear variable differential transducer (LVDT) driven by a lock-in amplifier (Model SR830, Stanford Research Systems, Sunnyvale, CA). The high field piezoelectric coefficients (*d*_33_) were determined from the slope of strain/field curves at the driving field.

#### Human arteries experiment

Human arteries were obtained from the biobank of Hubei Cancer Hospital. The protocol was sanctioned by the Medical Ethics Committee of Hubei Cancer hospital. The sections of diseased arteries were harvest and preserved in phosphate buffer. The artery specimens were immersed in water and supported by a sponge base to stand in a tank. After imaging, the imaged sections were pinpointed for hematoxylin-eosin(H&E)-stained histology examination.

## Additional Information

**How to cite this article**: Zhu, B. *et al*. New Potassium Sodium Niobate Single Crystal with Thickness-independent High-performance for Photoacoustic Angiography of Atherosclerotic Lesion. *Sci. Rep.* **6**, 39679; doi: 10.1038/srep39679 (2016).

**Publisher's note:** Springer Nature remains neutral with regard to jurisdictional claims in published maps and institutional affiliations.
